# Tetraploidization Altered Phenotypic Traits and Metabolite Profile of Java Ginseng (*Talinum paniculatum* (Jacq.) Gaertn.)

**DOI:** 10.3390/plants14030480

**Published:** 2025-02-06

**Authors:** Yingying Liu, Xiao Huang, Xinsheng Gao, Xiaofei Zhang, Huasun Huang, Weiguo Li, Yuanyuan Zhang

**Affiliations:** 1 Haikou Experimental Station, Chinese Academy of Tropical Agricultural Sciences, Haikou 571101, China; liu42315@163.com; 2 Rubber Research Institute, Chinese Academy of Tropical Agricultural Sciences, Haikou 571101, China

**Keywords:** tetraploidization, phenotypical changes, biomass, metabolites

## Abstract

Polyploidization is a beneficial technique for enhancing the biomass of and secondary metabolite concentrations in plants. Java ginseng (*Talinum paniculatum* (Jacq.) Gaertn.) can be used as an alternative source of nutrition and has both ornamental and medicinal value. To improve the biomass and content of medicinal ingredients, this study established an in vitro system that was used to induce polyploidy of java ginseng. Tetraploids were successfully produced by exposing the axillary buds to colchicine. The most favorable medium for inducing polyploidy was Murashige and Skoog medium devoid of hormonal substances, while immersing stem segments in a solution of 1–3 mg/mL colchicine for 48 h could achieve tetraploidy induction with a maximum rate of 18.03%. Tetraploids were distinguished from diploids by flow cytometry, with the tetraploids exhibiting darker and thicker leaves, bigger fruit and pollen, and larger stomata but lower stomatal density, while the aboveground biomass yield was reduced significantly compared with that of the diploids. Tetraploidization also altered the metabolite profile, with 22 metabolite concentrations being significantly increased (*p* < 0.05) and 74 metabolite concentrations being significantly decreased (*p* < 0.05) in the leaves of the tetraploids. The autotetraploid produced in this study could provide novel insights into artificial polyploid breeding and could be utilized as a germplasm to generate new polyploids.

## 1. Introduction

*Talinum paniculatum* (Jacq.) Gaertn., usually called java ginseng in southeast Asia, is a plant variety that is native to tropical and subtropical America. Today, it grows widely in the warm regions of the southern and northern hemispheres. The leaves of java ginseng contain high levels of ascorbic acid, protein, insoluble dietary fiber, magnesium, potassium, iron, and calcium, which can serve as an alternative nutritional source to diversify food [1,2]. Due to its extended flowering period and appealing, vibrant, and exquisite flowers, java ginseng is also utilized as an ornamental plant. Furthermore, java ginseng extracts contain saponins, flavonoids, tannins, triterpenes, sterols, and polyphenols endowed with antioxidative, antibacterial, and antifungal properties, making it a widely used plant in the treatment of numerous infirmities such as cancer, diabetes, hepatic ailments, leishmaniasis, and reproductive disorders [1,3,4,5,6,7]. Latest studies have suggested that ethanolic extracts of java ginseng may reduce cardiac structural damage [8,9]. So far, the utilization of java ginseng is still at the stage of the direct utilization of wild resources. Therefore, conducting targeted breeding to create new planting materials with higher biomass and medicinal components is of great significance for the utilization of java ginseng.

Polyploidization usually produces massive cells that result in an increased structural size and ascended levels of essential secondary metabolites. This process has become an effective approach to increase the biomass and levels of secondary metabolites in plants [10,11]. For instance, tetraploidization increased the leaf size, trichome density, and cannabidiol levels in *Cannabis sativa* [12] and improved the agronomic characteristics of and medicinal elements in *Anoectochilus formosanus* [13]. In *eucalyptus*, tetraploids induced by zygotic and somatic chromosome doubling augmented the oil gland size in the leaves [14,15]. Based on the same considerations, we assume that autotetraploidization may also alter the agronomic traits of java ginseng, including the aboveground biomass and secondary metabolite content, thereby enhancing its economic value.

Due to the lack of reports on the induction of tetraploid java ginseng, this study developed a rapid induction system for polyploid java ginseng using colchicine as a chromosomal doubling mutagen and determined the optimal induction treatment conditions. The phenotypic and agronomic traits of tetraploids and diploids of java ginseng were compared, and the metabolite content was analyzed through a metabolomics analysis. The tetraploids acquired in this study have the potential to increase the utility value of java ginseng and establish a foundation for triploid breeding in the next stage.

## 2. Results

### 2.1. Germination of Axillary Buds from Single Node Segments

**Figure 1:**
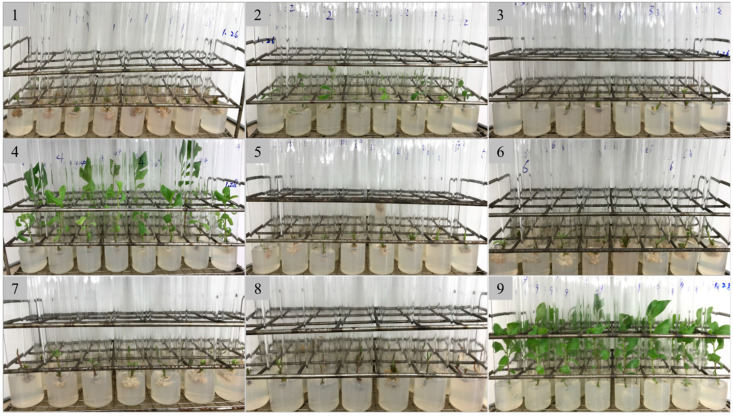
The regeneration of plants from axillary bud of java ginseng in different media. The numbers correspond to the media listed in the Table 1.

In the selection experiment with the axillary bud germination induction medium, all the treatments were achieved 100% germination of the axillary buds, while the growth vigor of the regenerated plants varied greatly (Figure 1). All the media yielded weak regenerated plantlets except for medium numbers four and nine. Specifically, medium numbers one, three, five, six, seven, and eight, without 6-BA, produced a callus at the lower end of the stem segment, resulting in rootless regenerated plants. Among the remaining three mediums, medium number nine, which contained no hormones, resulted in the most vigorous buds and roots, making it the optimal medium to induce polyploidy.

### 2.2. Tetraploid Induction and Ploidy Identification

**Figure 2:**
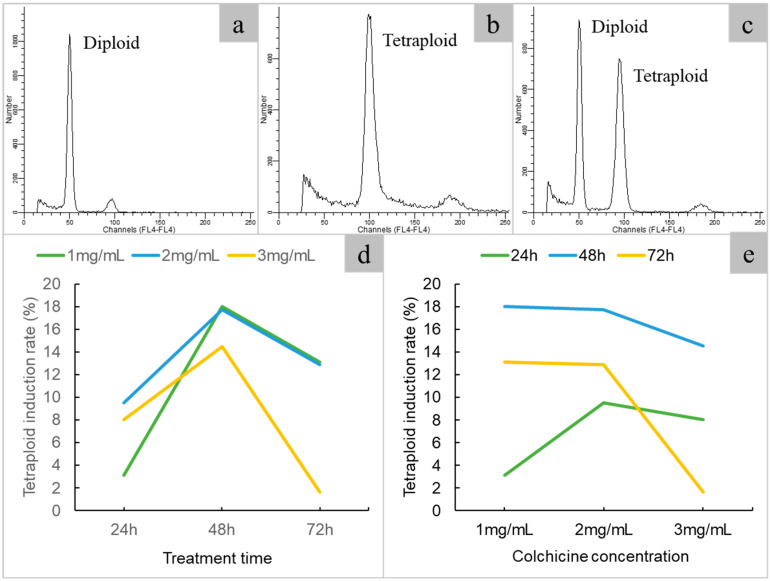
Flow cytometric analysis of different ploidy of java ginseng. (**a**) diploid; (**b**) tetraploid; (**c**) diploid and tetraploid; (**d**) effect of immersion time on polyploid induction; (**e**) effect of colchicine concentration on polyploid induction.

To induce polyploidy, a total of 658 single-node segments were treated with varying concentrations of colchicine and immersion times. A total of 398 plantlets were obtained, of which 61 were tetraploids and 73 were chimeras (Figure 2, Table 2). The optimal concentration of colchicine was found to be 1–3 mg/mL, with an ideal immersion time of 48 h. The highest induction rate achieved was 18.03% using 1 mg/mL colchicine for 48 h.

### 2.3. Phenotypical Changes in Tetraploids

**Figure 3:**
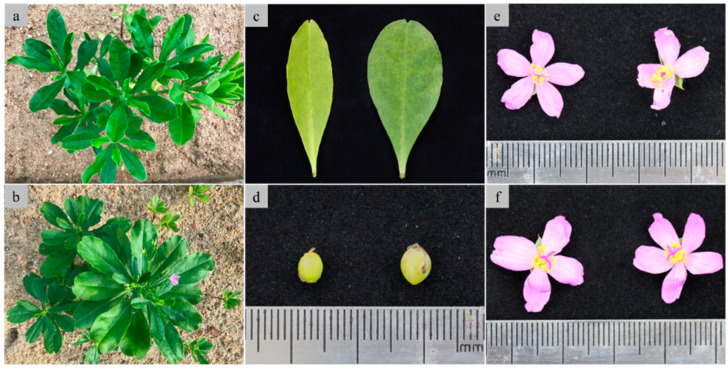
Phenotypic characteristics of diploid and tetraploid plants of java ginseng. (**a**) diploid plantlet; (**b**) tetraploid plantlet; (**c**) leaf of diploid (**left**) and tetraploid (**right**); (**d**) fruit of diploid (**left**) and tetraploid (**right**); (**e**) flower of diploid (with five petals, **left**) and tetraploid (with three petals, **right**); (**f**) flower of tetraploid with four petals (**left**) and five petals (**right**).

The influence of tetraploidization on the phenotypical characteristics was investigated. Compared with diploids, tetraploids had more leaves at the seedling stage (Figure 3a,b), darker green and more oval-shaped leaves (Figure 3c), bigger fruits (diameter of fruit: about 4 mm for tetraploid vs. about 3 mm for diploid) (Figure 3d), and slightly darker flowers. Additionally, a few three- and four- petal flowers were observed alongside the original five-petal flowers during the first time of bloom; these were not observed thereafter, and no abnormal flowers were observed in diploid plants, showing the significant impact of tetraploidization on the flower morphology of java ginseng at the early stage (Figure 3e,f).

**Figure 4:**
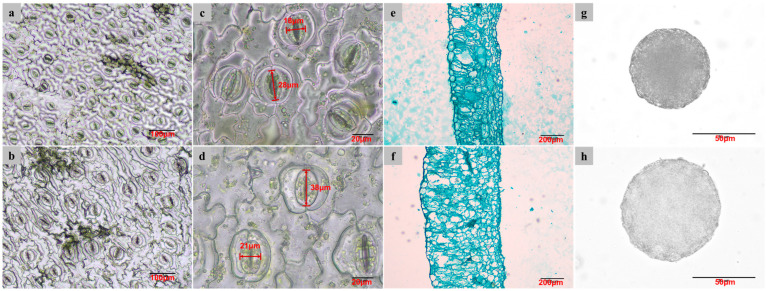
Stomatal and anatomical characteristics of diploid and tetraploid plants of java ginseng. (**a**) stomatal of diploid; (**b**) stomatal of tetraploid; (**c**) stomatal size of diploid; (**d**) stomatal size of tetraploid; (**e**) leaf anatomy of diploid; (**f**) leaf anatomy of tetraploid; (**g**) pollen of diploid; (**h**) pollen of tetraploid.

The differences in the stomatal, leaf anatomical, and pollen characteristics between diploids and tetraploids were analyzed (Table 3), and the changes related to ploidy level were investigated. ANOVA analysis showed that the stomatal length, stomatal width, stomatal density, leaf thickness, and pollen diameter were significantly different in diploid and tetraploid plantlets (Table 3). With tetraploidization, the stomatal length and width increased by 35.88% and 22.75% respectively (Figure 4a,b), while stomatal density decreased by 64.20% (Figure 4c,d). The average thickness of leaves increased by 87.88%, with larger leaf cells and looser tissues (Figure 4e,f), and the diameter of pollen grains increased by 29.97% (Figure 4g,h) with tetraploidization.

### 2.4. Comparison of Aboveground Biomass Between Diploid and Tetraploid

**Figure 5:**
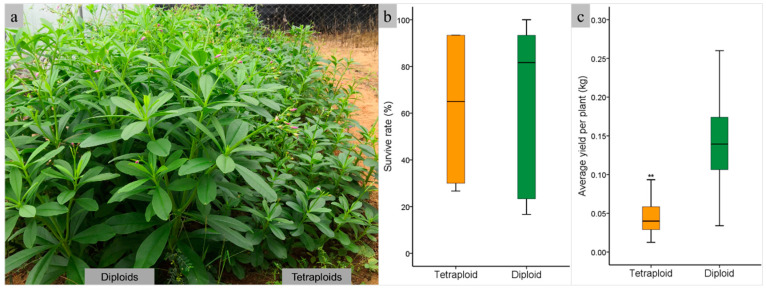
Comparison of aboveground biomass of different ploidy of java ginseng. (**a**) field planting of diploids and tetraploids; (**b**) box plots of survive rate of diploids and tetraploids; (**c**) box plots of aboveground biomass of diploids and tetraploids. ** represents significant difference (*p* < 0.01).

Diploids and tetraploids were planted in the field to evaluate the fresh weight yield of tetraploid plants. Plant growth performance showed that the height of tetraploids was much lower than that of diploids, which exhibited dwarfism (Figure 5a). The survival rates of diploids and tetraploids were 62.22% and 61.11%, respectively, with no significant difference (Figure 5b). The fresh weight of tetraploid plants was 0.04 kg per plant, significantly lower than that of diploids (*p* < 0.01), which had a fresh weight of 0.14 kg per plant (Figure 5c). The highest yields achieved of diploid and tetraploid were 0.25 kg and 0.09 kg per plant, respectively. The total fresh weights of the harvested aboveground biomass of diploids and tetraploids were 16.18 kg and 4.97 kg, respectively, with the same field area.

### 2.5. Metabolite Profile Changes in Tetraploids

**Figure 6:**
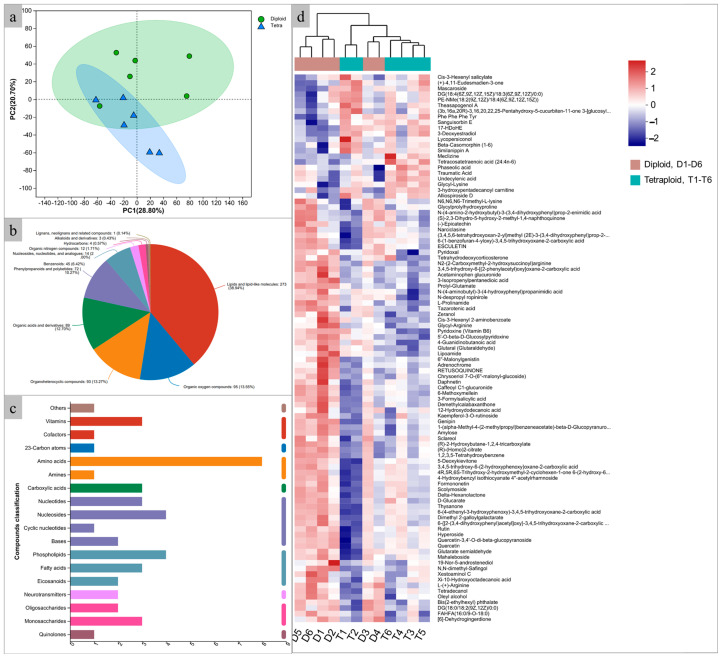
Metabolite analysis between diploids and induced tetraploids. (**a**) PCA of the relative difference in the metabolite profiles between diploids and tetraploids; (**b**) histogram of biochemical categories of the KEGG compound; (**c**) pie charts of the biochemical categories of the HMDB compound; (**d**) heat map of the relative difference in the metabolite profiles between diploid and tetraploid.

To explore the metabolic profile extensively, LC-MS was employed to identify the metabolites in java ginseng leaves. A PCA analysis showed that diploids and induced tetraploids were clearly separated, indicating substantial differences in their metabolite contents (Figure 6a). After database searches, a total of 892 metabolites were identified, of which 701 were identified in the HMDB database and classified into eleven categories, including lipids and lipid-like molecules (38.94%), organic oxygen compounds (13.55%), organoheterocyclic compounds (13.27%), organic acids and derivatives (12.70%), phenylpropanoids and polyketides (10.27%), benzenoids (6.42%), nucleosides, nucleotides and analogs (2.00%), organic nitrogen compounds (1.74%), hydrocarbons (0.57%), alkaloids and derivatives (0.43%), and lignans, neolignans, and related compounds (0.14%) (Figure 6b, Table S1). Of the 294 metabolites identified by the KEGG database, only 43 could be classified into seven categories, which included nucleic acids (23.26%), lipids (20.93%), peptides (16.28%), carbohydrates (11.63%), vitamins and cofactors (9.30%), organic acids (6.98%), hormones and transmitters (4.65%), antibiotics (4.65%), steroids (2.33%), and antibiotics (2.33%) (Figure 6c). Compared with diploids, the content of 22 metabolites, such as mascaroside (27.99% increase), undecylenic acid (15.19% increase), and tetracosatetraenoic acid (11.51% increase), were significantly increased (*p* < 0.05) in tetraploids, while the contents of 75 metabolites, such as formononetin (25.80% decrease), epicatechin (22.49% decrease), and 6-methoxymellein (18.84% decrease), were significantly decreased (*p* < 0.05) (Figure 6d, Table S2).

## 3. Discussion

Chemical reagents, particularly colchicine, are commonly used in plant polyploid breeding to double chromosomes [16,17,18]. In this study, induced tetraploids from axillary buds treated with colchicine were obtained, and the effects of the concentrations and exposure times were investigated for the first time. Eng and Ho [19] suggested that the concentration of colchicine used for tetraploid induction ranges from 0.05% to 1.0%, and the treatment time varies from hours to weeks with different methods in different horticultural plants. In *Thymus persicus*, the most efficient conditions for inducing polyploidy were treatment with 0.3% colchicine for 12 h, followed by 0.3% for 24 h, indicating that treatment time is flexible, as opposed to the concentration of colchicine [20]. In *cassava*, when the explants were treated for 2 days with different concentrations of colchicine (0.05–0.25 mg/mL), the death rate and tetraploid induction rate changed rapidly [21]. In this study, the optimum concentration of colchicine was 1–3 mg/mL, and the optimum immersion time was 48 h, revealing that immersion time is more important than the concentration of colchicine, which is inconsistent with the results reported in the aforementioned polyploid induction studies.

Polyploidization usually results in significant phenotypic changes in plants, such as cell enlargement, leaf thickening, changes in plant size and flower morphology, and alterations in fruit size [22]. Consequently, obtaining desirable traits through polyploidy is a popular method in plant breeding. In *Populus*, compared with the diploids, the tetraploids had larger and thicker leaves, larger but sparser stomata, fewer and shorter roots, and larger protoplasts [23,24]. In *Callisia fragrans*, the size of the leaf and flower nearly doubled when compared to the mother diploid plant [25]. In highbush blueberry, the stomatal sizes of the tetraploids were larger than those of the diploids, but with a reduced stomatal density [26]. In wallflower, the induced tetraploid showed a compact growth trend with shorter internodes and roots, bigger and purplish leaves, a prolonged flowering period, greater longevity, and bigger as well as a larger number of flowers, compared to diploid controls [27]. In *Manihot esculenta*, tetraploid plants showed better photosynthetic capacities than the original diploid plants [21]. Tetraploidization of java ginseng brought about significant changes in plant structure, including leaf color, leaf shape, leaf thickness, size of flowers and fruits, and number of petals. All these changes are similar to the results presented in the polyploid induction studies mentioned above.

Previous studies have proved that increases in cell size do not always translate to increases in the whole plant size, as the numbers of cell divisions in polyploids can be reduced, resulting in dwarfism in some species, including garlic [28] and *Populus* [24]. Currently, it is believed that plant hormones play an important role in the dwarfism caused by tetraploidization. In poplar [24], the contents of IAA and GA3 decrease and the contents of JA and ABA increase, resulting in the dwarfing of tetraploids. In apple [29], besides IAA, the decrease in the content of brassinosteroid (BR) is also one of the reasons. Dwarfism was also noted in this study, where the aboveground biomass of the tetraploid of java ginseng was significantly lower than that of diploids, implying that the increasing yield of java ginseng through chromosome doubling may need more tetraploidization testing. However, we did not find any differences in the content of the above-mentioned plant hormones between tetraploid and diploid plants. We speculated that the reason for this was that the collected leaves were mature and robust, had stopped growing, and had not yet aged. At this point, the content of growth hormones had decreased, while the content of aging hormones had not yet increased, making no difference in the content of plant hormones between diploids and tetraploids. This study is the first attempt to increase biomass by inducing tetraploid in java ginseng. Although the expected results were not obtained, it has reference significance for future java ginseng breeding. Moreover, further evaluation of the physiological performance of tetraploids, such as photosynthetic efficiency and root growth, is needed to elucidate the reasons for their low biomass.

Increasing the content of functional metabolites is crucial for breeding medicinal plants. Chromosome doubling has been successfully carried out in various plants to enhance their metabolite content. In *Thymus persicus* [20], tetraploids obtained through exposure of shoot tip segments to colchicine showed an increase of 69.73%, 42.76%, and 140.67% in the contents of betulinic acid, oleanolic acid, and ursolic acid, respectively, compared with diploids. In *Anoectochilus formosanus* [13], the leaf, stem, and whole plant of induced tetraploids produced more gastrodin and total flavonoid than the diploids. Artificial tetraploids of *Cannabis sativa* [12] were obtained by exposing axillary bud explants to oryzalin, resulting in an increase of 9% in the content of cannabidol in buds. In the induced tetraploids of *Digitalis lanata* [30], the content of digitoxin and digoxin increased by 1.73-fold and 1.61-fold, respectively, compared to the diploids. In this study, we observed similar results in the content of 22 metabolites in tetraploid leaves that were significantly increased compared with diploids, such as naphthofurans, fatty acyls, steroids and derivatives, prenol lipids, and so on. Among these metabolites, some have great medicinal values. For example, undecylenic acid, which increased by 15.19% in tetraploid compared with diploid, is a monounsaturated fatty acid and is currently in clinical use as a topical antifungal agent [31,32]; additionally, it has effective pro-apoptotic antitumor activity in vitro [33]. A metabolomics study of its leaves and the impact of polyploidization on metabolite content are of great significance for its potential development and utilization.

Autotetraploid can be used as a parent for triploid breeding. As shown in other plants such as poplar [34], rubber [35,36], and willow [37], triploids exhibit better vitality and stress resistance than diploids, and we anticipate that triploid java ginseng may also have similar advantages. Hybridization between tetraploids and diploids is a direct pathway for producing triploids, and the tetraploids obtained in our study will contribute to the next stage of java ginseng polyploid breeding.

## 4. Materials and Methods

### 4.1. Plant Materials

Healthy and strong stems of wild java ginseng from the same plant during its flowering time were sampled as explants in Danzhou, Hainan, China. Explants were soaked in 75% ethanol for 30 s, then immersed in a 0.1% HgCl_2_ solution for ten minutes with continuous shaking to sterilize and washed with aseptic water 4–5 times. Sterilized stems were divided into single node segments of 3–5 cm length and half inserted into solid MS medium [38]. The culture medium contained different concentrations of hormones for the induction of axillary bud germination. Before autoclaving the medium, the pH was adjusted to the range of 5.8–6.2. The entire cultivation process was carried out at 26 °C, under 2000 lx light and a 14/10-h light/dark cycle.

### 4.2. Culture Medium Selection and Polyploid Induction

To select the optimum culture medium for the induction of axillary bud germination, nine MS media containing different concentrations of 6-Benzylamino Purine (6-BA, 0 ppm, 1.5 ppm, and 3 ppm), 1-Naphthaleneacetic acid (NAA, 0 ppm, 0.5 ppm, and 1 ppm), and Kinetin (KT, 0 ppm, 0.25 ppm, and 0.5 ppm) were used (Table 1). After 20 days of incubation, the axillary bud germination rate was calculated, and the growth status of buds and roots was observed to evaluate the applicability of different culture media for polyploid induction.

The shoots from the medium selection stage were cut into 2–3 cm length single node segments and immediately immersed in liquid axillary bud germination induction medium with different concentrations of colchicine for varying lengths of time (24 h, 48 h, and 72 h). The liquid medium was dissolved with colchicine at different concentrations (1 mg/mL, 2 mg/mL, and 3 mg/mL) to induce chromosome doubling. After rinsing with water, the induced segments were transferred to the solid axillary bud germination induction medium (medium number nine) to regenerate plantlets. Each treatment used at least 30 segments, and each treatment was repeated.

### 4.3. Flow Cytometry for Ploidy Identification

Flow cytometry was employed to analyze the ploidy of the induced plants after 30 days of polyploid induction treatment. Fresh leaves of 1 cm^2^ size of each induced plantlet in the tube were sampled and placed in a plastic petri dish. We then added 500 µL of extraction buffer (Kobe, Hyōgo, Japan) and sectioned the specimens into splinters with a razor blade. The extracted nuclei were filtered through a filter with a pore size of 30 µm and then mixed with 1.6 mL staining buffer containing 10 ppm of 4′,6-diamidino-2-phenylindole (DAPI), Next, 50 µg/mL RNase (Kobe, Hyōgo, Japan) was added to the suspension to prevent staining of double-stranded RNA. After 30 s of incubation at room temperature, the samples were analyzed using CyFlow^®^Cube8 flow cytometry. The data obtained from flow cytometry were visualized using ModFit LT 3.1 (Cytonome Verity, LLC, Topsham, ME, USA). Wild type of java ginseng was used as an internal standard.

### 4.4. Leaf Stomatal Analysis

Forty days after planting, the stomatal characteristics, such as length, width, and density of mature leaves of tetraploid and diploid of java ginseng planted in a sandy bed in a greenhouse (sunlight, 25–30 °C), were measured using the nail polish method [39]. Clear nail polish was painted on the abaxial side of the leaves. After drying, the polish was peeled off together with the epidermis and flattened on glass slides. Three photos of stomata were captured under an Olympus CX43 (Olympus Corporation, Tokyo, Japan) microscope with 40× objective for each leaf for stomatal length and width measurements, while photos for stomatal density measurements were taken under a 20× microscope objective, using an LV2000 digital camera (Olympus Corporation, Tokyo, Japan). Three stomata were randomly selected in each photo to measure length and width in ImageJ 1.52a (https://imagej.net/ij/ (accessed on 13 August 2017)). Stomatal density was calculated in the same software. Overall, 27 datapoints for the stomata length and width, and nine datapoints for stomata density were obtained for each tetraploid and diploid, respectively. Three plants for each ploidy were random selected for leaf stomatal analysis.

### 4.5. Leaf Thickness Measurement

The thickness of the leaflets of tetraploids and diploids was measured using the paraffin section method. Mature leaves from diploid and tetraploid plants that were planted in a sandy bed in a greenhouse (sunlight, 25–30 °C), collected and cut into 0.5 cm^2^ pieces, and soaked in FAA fixing solution (V__Ethanol_:V__Formaldehyde_:V__Acetic acid_ = 90:5:5) for at least 24 h at 4 °C for fixation. After dehydration, vitrification, and embedding in paraffin, the leaves were sectioned into 10–12 µm samples and stained with Fast green. They were then surveyed under an Olympus CX43 (Olympus Corporation, Tokyo, Japan) microscope. Photographs were captured with a 20× objective for each leaf using an LV2000 digital camera. Thickness measurements were taken using ImageJ software. In total, 27 datapoints of leaf thickness were collected for each tetraploid and diploid, respectively. Three plants for each ploidy were randomly selected for leaf thickness analysis.

### 4.6. Pollen Size Measurement

Anthers from bloomed male flowers were picked out and crushed with tweezers on a slide and stained with acetic acid magenta staining solution. Slides containing pollen were observed and photographed using the same microscope and photography system as that use for leaf thickness measurement. Pollen diameters were measured using ImageJ software. In total, at least 50 pollen grains from tree flowers were measured for both diploids and tetraploids.

### 4.7. Aboveground Biomass of Plantings

To compare the yield of aboveground biomass of diploid and tetraploid, four artificially induced tetraploid and four parental diploid plants were randomly selected as clones and planted in the field by stem cutting. The land is red brick soil with an average environmental temperature of around 27 °C. The plants were watered once a week. Each clone was planted with 30 plants in the form of 6 rows of 5 plants, with a plant spacing and row spacing of 5 cm and 10 cm, respectively. After 60 days of planting, the survival rate was counted, and the aboveground biomass was harvested. The fresh weight per row was weighed, and the average yield per plant was calculated by dividing the fresh weight per row by the number of plants per row.

### 4.8. Liquid Chromatography-Mass Spectrometry Analysis

Liquid Chromatography-Mass Spectrometry (LC-MS) analysis was used to compare the metabolite content of the mature leaves of six diploid plants (D1–D6) and six tetraploid plants (T1–T6). Fresh leaves with the same position and growth state on the stems of diploids and tetraploids were sampled. After washing with water, clean leaves were weighed and frozen in liquid nitrogen, then stored in a −80 °C refrigerator. Sampled leaves were then sent to Shanghai Majorbio Bio-Pharm Technology Co., Ltd. (Shanghai, China) for metabolite extraction and LC-MS analysis. The process of extracting metabolites was carried out in accordance with the company’s internal technical standards. Briefly, 50 mg solid sample was accurately weighed, and the metabolites were extracted using a 400 µL methanol:water (4:1, *v*/*v*) solution with 0.02 mg/mL L-2-chlorophenylalanine as an internal standard. The mixture was allowed to settle at −10 °C and treated by a high throughput tissue crusher Wonbio-96c (Shanghai Wanbo Biotechnology Co., Ltd., Shanghai, China) at 50 Hz for 6 min, followed by ultrasound at 40 kHz for 30 min at 5 °C. The samples were placed at −20 °C for 30 min to precipitate proteins. After centrifugation at 13,000× g at 4 °C for 15 min, the supernatant was carefully transferred to sample vials for LC-MS/MS analysis. As a part of the system conditioning and quality control process, a pooled quality control sample (QC) was prepared by mixing equal volumes of all samples. The QC samples were disposed of and tested in the same manner as the analytic samples. During instrument analysis, we inserted a QC sample every eight analytical samples to assess the repeatability of the entire analysis process.

The instrument platform for LC-MS analysis was the UHPLC-Q Exactive system of Thermo Fisher Scientific. Chromatographic conditions: 2 μL of sample was separated by a BEH C18 column (100 mm × 2.1 mm i.d., 1.7 μm; Waters, Milford, MA, USA) and then entered into mass spectrometry detection. The mobile phases consisted of 0.1% formic acid in water (solvent A) and 0.1% formic acid in acetonitrile:isopropanol (1:1, *v*/*v*) (solvent B). The solvent gradient changed according to the following conditions: from 0 to 3 min, 0% B to 20% B; from 3 to 9 min, 20% B to 60% B; from 9 to 11 min, 60% B to 100% B; from 11 to 13.5 min, 100% B to 100% B; from 13.5 to 13.6 min, 100% B to 0% B; from 13.6 to 16 min, and 0% B to 0% B to equilibrate the systems. The sample injection volume was 20 µL and the flow rate was set to 0.4 mL/min. The column temperature was maintained at 40 °C. During the period of analysis, all these samples were stored at 4 °C. 

The mass spectrometric data were collected using a Thermo UHPLC-Q Exactive Mass Spectrometer (Thermo Fisher Scientific Inc., Bremen, Germany) equipped with an electrospray ionization (ESI) source operating in either positive or negative ion mode. The ion-spray voltage floating was −2800 V in negative mode and 3500 V in positive mode, respectively; normalized collision energy, 20–40–60 V rolling for MS/MS. Ion source temperature and solvent removal temperature are 120 °C and 500 °C respectively, the carrier gas flow rate was 900 L/h, mass spectrum scanning range was 50–1000 *m*/*z*, and resolution was 30,000. After the mass spectrometry detection had been completed, the raw data of LC/MS were preprocessed by Progenesis QI 3.0 (Waters Corporation, Milford, MA, USA) software, and a three-dimensional data matrix in CSV format was exported. The information in this three-dimensional matrix included sample information, metabolite name, and mass spectral response intensity. Internal standard peaks, as well as any known false positive peaks (including noise, column bleed, and derivatized reagent peaks), were removed from the data matrix and peak pooled. At the same time, the metabolites were searched and identified; the main database was the HMDB (http://www.hmdb.ca/ (accessed on 27 August 2024)), Metlin (https://metlin.scripps.edu/ (accessed on 28 August 2024)) and Majorbio Database.

The data after the database search were uploaded to the Majorbio cloud platform (https://cloud.majorbio.com (accessed on 28 August 2024)) for data analysis. Metabolic features detected in at least 80% of any set of samples were retained. After filtering, minimum metabolite values were imputed for specific samples in which the metabolite levels fell below the lower limit of quantitation and each metabolic feature was normalized by sum. To reduce the errors caused by sample preparation and instrument instability, the response intensity of the sample mass spectrum peaks was normalized by the sum normalization method, and a normalized data matrix was obtained. At the same time, variables with relative standard deviation (RSD) > 30% of QC samples were removed, and log10 logarithmization was performed to obtain the final data matrix for subsequent analysis.

Next, we performed a variance analysis on the matrix file after data preprocessing. The R package ropls (Version 1.6.2) performed principal component analysis (PCA) and orthogonal least partial squares discriminant analysis (OPLS-DA) and used 7-cycle interactive validation to evaluate the stability of the model. In addition, student’s *t*-test and fold difference analysis were performed. The selection of significantly different metabolites was determined based on the variable importance in the Projeciton (VIP) obtained by the OPLS-DA model and the *p*-value of student’s *t*-test; VIP > 1 and *p* < 0.05 indicated significantly different metabolites.

### 4.9. Data and Statistical Analysis

One-way ANOVA and LSD post hoc multiple comparison analyses were performed for the length, width, and density of stomata, as well as leaf thickness, pollen size, and aboveground biomass, using SPSS Statistics 22.0 software (IBM Corp., Armonk, NY, USA).

## 5. Conclusions

This study demonstrated that java ginseng node segments were suitable explants for tetraploid induction. The optimal conditions for tetraploid induction were segments immersed in a liquid medium containing 1–2 mg/mL colchicine for 48 h. Compared with diploids, tetraploids of java ginseng exhibited larger flowers, fruits, and pollen, as well as larger but fewer stomata. The aboveground biomass of tetraploids was significantly decreased compared with diploids. In the leaves of tetraploids, the contents of 22 metabolites increased significantly (*p* < 0.05), while those of 74 metabolites decreased significantly (*p* < 0.05). This study investigated polyploid induction in java ginseng for the first time and compared differences in artificially induced tetraploid and diploid in multiple aspects.

## Figures and Tables

**Table 1:** Effects of hormone concentrations on axillary bud germination of java ginseng.

Medium	Hormone Concentrations (ppm)		
	6-BA	NAA	KT
1	3	1	0
2	0	0.5	0.5
3	3	0	0.5
4	0	1	0.25
5	1.5	1	0.5
6	3	0.5	0.25
7	1.5	0.5	0
8	1.5	0	0.25
9	0	0	0

**Table 2:** Tetraploid induction rate of java ginseng with different concentrations of colchicine medium and immersion times.

Treatment Time (h)	Colchicine Concentration (mg/mL)	No. of Explants	No. of Plantlets	No. of Tetraploids	No. of Chimeras	Tetraploid Induction Rates ± SE (%)
24	1	64	60	2	8	3.13 ± 0.10
24	2	63	59	6	9	9.52 ± 4.62
24	3	62	44	5	11	8.06 ± 12.90
48	1	61	56	11	7	18.03 ± 6.82
48	2	62	52	11	9	17.74 ± 1.04
48	3	62	31	9	8	14.52 ± 3.65
72	1	61	45	8	7	13.11 ± 1.88
72	2	62	34	8	10	12.9 ± 5.31
72	3	61	17	1	4	1.64 ± 1.72

**Table 3:** Comparative analysis of the characteristics of diploid and tetraploid of java ginseng.

Traits	Diploids	Tetraploids
Stomatal length (µm)	30.01 ± 3.45 b	40.78 ± 4.76 a
Stomatal width (µm)	18.59 ± 2.33 b	22.82 ± 3.65 a
Stomatal density (mm^−2^)	149.93 ± 31.79 a	53.68 ± 10.36 b
Leaf thickness (mm)	0.33 ± 0.04 b	0.62 ± 0.07 a
Pollen diameter (μm)	65.36 ± 1.92 b	84.95 ± 5.69 a

Each value represents the mean ± SD of two experiments that involved at least 27 replicates. The different letters followed by values within the same row indicate extremely significantly differences (*p* < 0.01).

## Data Availability

Data is contained within the article or Supplementary Materials.

## Supplementary Materials

The following supporting information can be downloaded at: https://www.mdpi.com/article/10.3390/plants14030480/s1, Table S1: Biochemical categories of the KEGG compound; Table S2: Relative difference in the metabolite profiles between diploids and tetraploids.
